# The Future of Oncology in Psychiatric Medications

**DOI:** 10.3390/jcm14176003

**Published:** 2025-08-25

**Authors:** Napoleon Waszkiewicz

**Affiliations:** Department of Psychiatry, Medical University of Białystok, 15-272 Białystok, Poland; napoleon.waszkiewicz@umb.edu.pl; Tel.: +48-608-888-796

**Keywords:** oncology, psychiatry, medication, tumor, cancer, depression, psychosis, bipolar, stress

## Abstract

Recent years have provided numerous reports on the mechanisms of action of psychiatric medications (antidepressants, antipsychotics, mood stabilizers, and antidementia drugs) that directly inhibit the growth of cancer cells, as well as on their indirect effects on the psyche and immune system, and their supportive effects on chemotherapeutic agents. The mechanisms of the anticancer activity of psychiatric drugs include inhibition of dopamine and N-methyl-D-aspartate receptors that work via signaling pathways (PI3K/AKT/mTOR/NF-κB, ERK, Wnt/ß-catenin, and bcl2), metabolic pathways (ornithine decarboxylase, intracellular cholesterol transport, lysosomal enzymes, and glycolysis), autophagy, Ca^2+^-dependent signaling cascades, and various other proteins (actin-related protein complex, sirtuin 1, p21, p53, etc.). The anticancer potential of psychiatric drugs seems to be extremely broad, and the most extensive anticancer literature has been reported on antidepressants (fluoxetine, amitriptyline, imipramine, mirtazapine, and St John’s Wort) and antipsychotics (chlorpromazine, pimozide, thioridazine, and trifluoperazine). Among mood stabilizers, lithium and valproates have the largest body of literature. Among antidementia drugs, memantine has documented anticancer effects, while there is limited evidence for galantamine. Of the new psychiatric substances, the antipsychotic drug brexpiprazole and the antidepressant vortioxetine have a very interesting body of literature regarding glioblastoma, based on in vitro and in vivo animal survival studies. Their use in brain tumors and metastases is particularly compelling, as these substances readily cross the blood–brain barrier (BBB). Moreover, the synergistic effect of psychiatric drugs with traditional cancer treatment seems to be extremely important in the fight against chemo- and radio-resistance of tumors. Although there are some studies describing the possible carcinogenic effects of psychiatric drugs in animals, the anticancer effect seems to be extremely significant, especially in combination treatment with radio/chemotherapy. The emerging evidence supporting the anticancer properties of psychiatric drugs presents an exciting frontier in oncology. The anticancer properties of psychiatric drugs may prove particularly useful in the period between chemotherapy and radiotherapy sessions to maintain the tumor-inhibitory effect. While further research is necessary to elucidate the mechanisms, clinical implications, dose-dependence of the effect, and clear guidelines for the use of psychiatric medications in cancer therapy, the potential for these commonly prescribed medications to contribute to cancer treatment enhances their value in the management of patients facing the dual challenges of mental health and cancer.

## 1. Introduction

Although the field of psycho-oncology is relatively new, the first collaboration between psychiatry, surgical oncology, and radiotherapy took place in the mid-1950s, marking the beginning of psycho-oncology. The role of a psychiatrist specializing in the treatment of cancer patients, a psychosocial oncologist or psycho-oncologist, encompasses the entire spectrum of care, from preventative care to end-of-life care. The psycho-oncologist, as a member of the multidisciplinary treatment team, provides specific care, addressing cancer-related issues that arise throughout the course of the disease. They bring expertise in combining targeted therapeutic strategies with pharmacological interventions to address the multifaceted symptoms of oncological patients [1].

The psychological and psychiatric burden of cancer is profound, affecting not only the patients but also their families and caregivers [2]. Studies indicate that the lifetime prevalence of any mental disorder in cancer patients is more than 56%, and the point-prevalence during cancer is more than 38% (19% for adjustment disorders, 16% for depression, and 10% for anxiety disorders) [2,3]. The highest levels of distress are noticed in patients with female genital cancers and pancreatic cancer [4]. This may be related to poor cancer prognosis, but also to high levels of inflammatory factors, as shown by the relationship between high levels of interleukin-6 and the severity of depression symptoms in pancreatic cancer [5]. As increased incidence rates of brain and lung cancers among first-time psychiatric patients can be found, the psychiatric condition may represent one of the earliest manifestations of paraneoplastic potential of cancer immunology [5]. Patients who received all three modalities (chemotherapy, radiotherapy, and surgery) and those treated with alkylating agent chemotherapeutics had the highest burden of psychiatric disorders, whereas those treated with kinase inhibitors had the lowest burden [5]. Even among cancer survivors, the prevalence of mental disorders remains high: 34% of people experience sleep disorders, 24% experience anxiety, and 23% experience depression [4]. Spouses, parents, and siblings of cancer patients can also experience increased rates of depression, anxiety, and other psychiatric disorders. This impact is often tied to the stress of caregiving, emotional distress related to the diagnosis and treatment, and concerns about the future [6]. Cancer and mental health patients die prematurely and are more likely to self-harm [5,6,7]. The risk of suicide is 20% higher in patients with cancer compared with the general population, whereas about 59% of terminally ill patients who request assisted suicide are depressed [5,8].

People with severe mental illness (SMI) experience more than 30% higher cancer mortality rates compared with the general population, despite often having lower cancer incidence [9]. The higher cancer mortality in the psychiatric population is likely due to a combination of factors, including delayed diagnosis, reduced access to cancer screening and treatment, and poorer overall health among individuals with SMI [9]. However, it has also been suggested that the lower incidence of cancers of old age in SMI might be due to premature aging and death in this population [9]. Additionally, it has been found that the prevalence of certain cancers, particularly lung and breast cancer, is significantly higher in individuals with mental health disorders. This increased risk is attributed to various factors, including lifestyle choices such as smoking, poor diet, and medication side effects [9]. Data suggest that psychosocial stress could also affect cancer incidence and/or mortality, and potential candidate mechanisms include the neuroendocrine signaling pathways initiated by stress that affect antitumor immune cells, promoting an immunosuppressive and pro-tumor microenvironment [10].

Mental and physical illnesses are closely connected and often influence each other bidirectionally [7,11]. Mental disorders such as depression and anxiety can increase the risk of developing chronic physical conditions such as cardiovascular disease, diabetes, and gastrointestinal problems because of biological (e.g., inflammation and hormonal changes) and behavioral (e.g., insufficient self-care and substance abuse) factors. Conversely, chronic physical illnesses can lead to or exacerbate mental health problems through stress, lifestyle changes, and neurobiological factors [11].

After reviewing the main problem of the prevalence of psychiatric problems in cancer patients, I address the main advantages and disadvantages of psychiatric treatment in oncological diseases, focusing on the latest works describing the pharmacological effects of treatment observed in recent years. Therefore, the primary focus of this article is the repurposing potential of psychiatric drugs in oncology, and the psycho-oncological context is provided as a work background. Additionally, I suggest future research avenues for potential therapies for both oncological and psychiatric diseases simultaneously.

## 2. Methods

A literature search was conducted in PubMed, Scopus and Web of Science databases using keywords: ‘depression’, ‘schizophrenia’, ‘bipolar disorder’, ‘dementia’, ‘psychiatric disorders’, ‘psychiatric diseases’, ‘psychiatric drugs’, ‘antidepressants’, ‘antipsychotics’, ‘normothymics’, ‘mood stabilizers’, ‘antidementia drugs’, ‘cancer’, ‘cancer treatment’, ‘oncology’, and ‘repurposing’, as well as combinations of these terms. Since this work is of the perspective type, no strict inclusion criteria were established for the selection of articles. Both original research and review articles published between 2006 and 2025 were considered during the initial screening studies, based on titles and abstracts, while conference abstracts were excluded. Studies not written in English were also omitted. The relevant articles were then included with the intention to cover the widest possible spectrum of treatment of oncological diseases with psychiatric medications.

## 3. Advantages and Disadvantages of Using Psychiatric Drugs in Oncology

### 3.1. Disadvantages

Psychiatric medications in cancer treatment pose several disadvantages that may affect patient outcomes and overall care. Psychiatric medications often come with a range of side effects, such as sedation, dizziness, and gastrointestinal disturbances. These can mimic the physical side effects of cancer treatments such as chemotherapy and radiation, making it more challenging for patients to tolerate their cancer therapies [7]. In addition, cancer patients typically take multiple medications, which increases the risk of drug interactions. Psychiatric medications can interact with chemotherapy agents (e.g., tamoxifen and antidepressants metabolized by the same cytochrome CYP2D6), potentially diminishing their effectiveness or heightening their toxicity [12]. Some psychiatric medications, such as benzodiazepines, can impair cognitive function, which may be particularly problematic for cancer patients who are already facing cognitive challenges because of their illness or treatment [7]. Other complications of psychiatric treatment in cancer patients include the stigma associated with mental health treatment that may lead some patients to avoid necessary psychiatric care. The complexity of managing multiple medications can also result in non-compliance, whereas incorporating psychiatric medications into a treatment regimen may delay the initiation of essential cancer therapies, which can be particularly critical in aggressive cancers where prompt treatment is vital [13]. Some psychiatric medications, such as benzodiazepines or opioids, also carry a risk of dependence syndrome, which can complicate the general treatment processes because of withdrawal symptoms [7].

Although psychiatric medications can alleviate symptoms of anxiety and depression, they do not address the underlying psychosocial issues that cancer patients often face, such as fear of relapse and relationship stressors [14]. Therefore, given the complex interplay between cancer and mental health, an integrative approach is often recommended. By combining pharmacological treatments with psychotherapy, such as cognitive–behavioral therapy (CBT), individual meaning-centered psychotherapy (IMCP), and Mindfulness-Based Cognitive Therapy (MBCT), we can obtain much better outcomes. Such integrative models not only address the psychological aspects of cancer by relieving stress but also empower patients to engage actively in their care, strengthening immunity and improving their quality of life [14].

In addition to depression treatment, antidepressants are used to treat anxiety disorders, including generalized anxiety disorder and obsessive–compulsive disorder, etc., but they are also used off-label with a special focus on treating eating disorders, sleep problems, smoking cessation, and chronic pain [7,15]. Although antidepressants are among the most commonly used drugs in the world and are highly effective [16], they are not without risks. Common side effects such as gastrointestinal disturbances, insomnia, sexual dysfunction, and the potential for drug interactions must be considered, especially in oncology patients who are often on multiple medications [7,17]. Therefore, close monitoring by healthcare professionals is essential to mitigate these risks.

### 3.2. Advantages: Psychiatric Drugs as a New Frontier in Oncology

**Figure 1:**
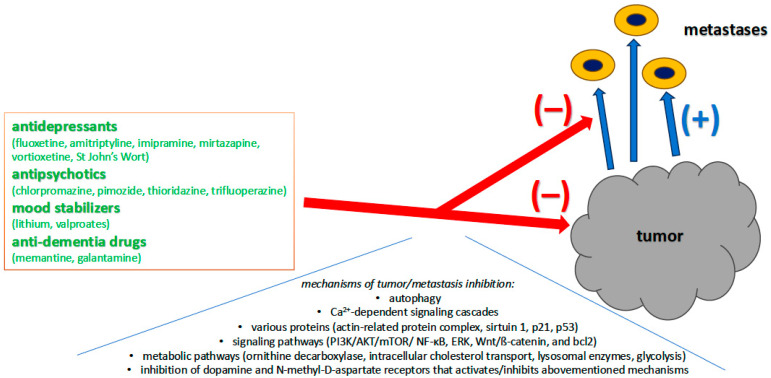
Psychiatric drugs and their mechanisms of action in inhibiting the growth and metastasis of cancer cells. Red arrows—inhibition, blue arrows—induction.

The primary role of using psychiatric medications in oncology patients is the frequent mental disorders occurring in this population [7]. In recent years, a growing body of research has begun to uncover a surprising and promising link between psychiatric medications and cancer treatment [18,19,20,21,22,23,24,25,26,27]. Several psychiatric drugs traditionally used to manage mental health disorders, such as depression, schizophrenia, bipolar disorder, and dementia, have demonstrated potential anticancer properties in preclinical and clinical studies (Table 1, Table 2 and Table 3; Figure 1). However, there are also studies available describing the procarcinogenic effect of psychotropic drugs, especially pointing out the carcinogenicity of new generation (atypical) antipsychotics and anticonvulsants [28]. In female schizophrenia patients, an elevated incidence of breast cancer has been found, which might be due to the elevated serum prolactin levels [26]. In patients treated with clozapine, the hematological side effects were linked to an increased risk of acute myeloid leukemia [26]. On the other hand, psychiatric patients more often smoke cigarettes (~80% of schizophrenia patients) and drink alcohol than the general population, but there is no noted increase in the incidence of cancer in this population, despite the significant carcinogenic properties of these substances [26]. In fact, there is even a decrease in gastrointestinal and brain tumors in this population [26].

Recent years have seen unsatisfactory progress in the development of oncological treatment. This is especially evident in the case of glioblastoma, the deadliest primary brain cancer, in which targeted therapies have been largely unsuccessful, partly because of the BBB limiting tumor treatment accessibility [23]. The solution to the challenging situation in oncology may lie in the assistance of another field of medicine, namely psychiatry, and the drugs used there, which easily penetrate the BBB [22,23,24,25,26]. The BBB allows the passage of low molecular weight particles of 400 to 600 Daltons [26]. Therefore, psychiatric medications, traditionally used to treat mental health disorders such as depression, schizophrenia, bipolar disorder, and dementia, are increasingly being investigated for their potential anticancer properties. This repurposing of psychotropic drugs offers a promising avenue for oncology, especially given their well-characterized pharmacokinetics and safety profiles [18,24,29,30].

**Table 1:** The effect of antidepressive drugs on various cancers [7,21,22,23,24,25,31]. The literature is primarily based on in vitro studies of cancer cell lines and in vivo studies in animals that were implanted with cancer cells and subsequently studied after tumor formation. The effects of psychiatric drugs on human cancers in vivo (CT, clinical trials) are described in Section 4.

Antidepressive Drug		Psychiatric Disorder Treated	Cancer Treated	Anticancer Mechanism of Psychiatric Drugs
selective serotonin reuptake inhibitors (SSRIs)	fluoxetine	- depression - generalized anxiety disorder - obsessive–compulsive disorder - panic disorder - post-traumatic stress disorder - social anxiety disorder - bulimia nervosa (fluoxetine)	- colon, gastric, liver, non-small cell lung, ovarian cancer, Burkitt lymphoma, glioma, lymphoma, neuroblastoma	- increased apoptosis/autophagy through mitochondrial dysfunction, ROS, Ca^2+^, NGF receptor, Fas receptor, caspase-7, cyclins, p21, p53 - modification of signaling pathways such as JNK/c-Jun, PI3K/AKT/mTOR, NK-κB, AMPK - antiproliferative effects that regulate tumor growth and metastasis through cell cycle regulators, inhibition of MMP-9, VEGF, Eag1 channels, inhibition of FoxM1-associated DNA repairing, enhanced Lin-7 C expression - inhibition of tumor development by the immune response through cytokines such as TNF, IL-12, IFN, and chemokines
	paroxetine		- breast, colon cancer, osteosarcoma	
	sertraline		- gastric cancer, stem cell cancer	
	citalopram		- glioma, glioblastoma, hepatocellular carcinoma, non-small cell lung cancer	
tricyclic antidepressants (TCAs)	amitryptyline		- liver, lung cancer, osteosarcoma	
	imipramine		- breast, colorectal, head and neck, prostate cancer, glioblastoma, glioma, leukemia	
serotonin–norepinephrine reuptake inhibitors (SNRIs)	duloxetine		- pancreatic cancer	
tetracyclic antidepressants	mirtazapine		- glioblastoma, osteosarcoma	
monoamine oxidase inhibitor (MAOI)	phenelzine		- prostate cancer	
serotonin modulator and stimulator (SMS)	vortioxetine	- depression	- gastric cancer, glioblastoma	
herbs	St. John’s Wort	- depression	- breast, colorectal cancer, leukemia	

**Table 2:** The effects of antipsychotic drugs on various cancers [7,18,19,20,21,26,27,32]. The literature is primarily based on in vitro studies of cancer cell lines and in vivo studies in animals that were implanted with cancer cells and subsequently studied after tumor formation. The effects of psychiatric drugs on human cancers in vivo (CT, clinical trials) are described in Section 4.

Antipsychotic Drug	Psychiatric Disorder Treated	Cancer Treated	Anticancer Mechanism of Psychiatric Drugs
aripiprazole	- schizophrenia - schizoaffective disorder - delusional disorder - bipolar disorder - delirium - delusional disorder - psychotic depression - substance-related psychosis	- breast, colorectal, gastric, head and neck, hepatocellular, non-small cell lung, pancreatic cancer, glioblastoma, melanoma	- blockade/inhibition of dopamine-D2 receptor results in cytotoxic effects through activation of the integrated stress response - modification of signaling pathways such as PI3K,/AKT/mTOR/MDMA2, NF-κB, ERK, NRF2, Wnt/ß-catenin, bcl2 - modification of metabolic pathways such as ornithine decarboxylase, intracellular cholesterol transport, lysosomal acid sphingomyelinase, estrogen receptor alpha, and glycolysis (by GSK3) - deregulation of proteins such as actin-related protein 2/3 complex, REST, TGF-ß, SIRT - increased autophagy/apoptosis through phosphorylation of STAT3, MMP-9, caspase-3, c-Myc, Bcl-2, BAX, KLF5, FOXO3 transcription factor - modification of Ca^2+^-dependent signaling cascades through calmodulin and phospholipase in the reticulum - other suppressed/inhibited mechanisms/proteins such as gene CD133 and SOX2, Yes-associated protein, ubiquitin carboxyl-terminal hydrolase, CDK2, K-Ras protein, topoisomerase, Na^+^/K^−^/ATPase pump, p53 protein, PAK4, TCTP, SNAI1, NUPR1
brexpiprazole		- breast, colorectal, non-small cell lung, pancreatic cancer, glioblastoma, glioma	
chlorpromazine		- breast, colorectal, gingival, laryngeal, liver, lung, ovarian, pancreatic, skin cancer, Ehrlich ascites tumor, fibro/sarcoma, glioblastoma, glioma, leukemia, lymphoblastoma, mastocytoma, medulloblastoma, melanoma, sarcoma	
chlorprothixene		- acute myeloid leukemia, medulloblastoma, prostatic cancer	
clozapine		- gastric, non-small cell lung, prostatic cancer, melanoma	
fluphenazine		- breast, colorectal, liver, non-small and small cell lung, ovarian, pancreatic, prostatic cancer, glioma, leukemia, melanoma	
flupenthixol		- breast, colorectal, lung cancer, melanoma	
fluspirilene		- breast, colorectal, liver, lung, ovarian, prostatic, renal cancer, glioblastoma, leukemia, melanoma	
haloperidol		- breast, colorectal, lung, pancreatic cancer, Ehrlich ascites tumor, glioblastoma, glioma, leukemia, melanoma, uterine sarcoma	
iloperidone		- breast, colorectal cancer, glioblastoma	
olanzapine		- breast, lung, pancreatic cancer, glioblastoma, glioma, lymphoblastoma	
penfluridol		- breast, colorectal, esophageal, gallbladder, liver, lung, pancreatic, prostate, renal cancer, glioblastoma, glioma, leukemia, melanoma	
perphenazine		- breast, colorectal, endometrial, liver, lung, pancreatic, prostate, skin cancer, glioblastoma, glioma, leukemia, lymphoma, melanoma, sarcoma	
pimavanserin		- esophageal, pancreatic cancer, glioblastoma	
pimozide		- breast, colorectal, liver, lung, pancreatic, prostate cancer, glioblastoma, glioma, leukemia, lymphoma, melanoma, multiple myeloma, osteosarcoma, retinoblastoma	
prochlorperazine		- breast, colorectal, lung, ovarian, prostate cancer, glioblastoma, leukemia, melanoma	
quetiapine		- gastric, liver cancer, glioblastoma	
risperidone		- colorectal, gastric, prostate cancer, glioblastoma, glioma, uterine sarcoma	
sertindole		- breast, gastric cancer, leukemia	
spiperone		- breast, colorectal, gastric cancer, glioblastoma, glioma	
sulpiryde		- breast cancer	
thioridazine		- breast, cervical, colorectal, endometrial, esophageal, gastric, liver, lung, nasopharyngeal, pancreatic, prostate, skin, testis cancer, glioblastoma, glioma, leukemia, lymphoma, medulloblastoma, melanoma, osteosarcoma	
trifluoperazine		- breast, cervical, colorectal, endometrial, esophageal, gastric, liver, lung, nasopharyngeal, pancreatic, peripheral nerve sheath, prostate, renal, skin, testis, urinary bladder cancer, glioblastoma, glioma, leukemia, lymphoma, melanoma, multiple myeloma	
zuclopenthixol		- breast, gastric, ovarian cancer	

**Table 3:** The effect of mood stabilizers and antidementia drugs on various cancers [29,30,33,34,35]. The literature is primarily based on in vitro studies of cancer cell lines and in vivo studies in animals that were implanted with cancer cells and subsequently studied after tumor formation. The effects of psychiatric drugs on human cancers in vivo (CT, clinical trials) are described in Section 4.

Psychiatric Drug		Psychiatric Disorder Treated	Cancer Treated	Anticancer Mechanism of Psychiatric Drugs
mood stabilizers	lithium	- bipolar disorder	- brain, liver, ovarian, prostate, thyroid cancer, glioblastoma, leukemia	- enhanced antitumor properties of anticancer drugs - induced apoptosis and autophagy, cell cycle arrest, inhibited tumor proliferation, invasion, and metastasis, as well as immunological damage through inhibition of coupling of β-adrenergic and muscarinic receptors to G proteins, BPNT1, GSK-3β, MYCN, modification of WNT/β-catenin, CREB, and immunomodulation of lymphokine killer cells
	valproates		- breast, cervical, gastric, head and neck, lung, prostate, skin, thyroid cancer, glioblastoma, glioma, hepatocellular carcinoma, leukemia, lymphoma, myeloma	- decreased resistance to chemotherapy through stimulation of silenced tumor inhibitor genes and inhibition of HDAC - increased apoptosis through CCAAT-enhancer-binding protein alpha/sterol regulatory element-binding protein 1 - modified tumor/cell proliferation through PKM2, p21, Wnt 1,2, cyclin D3, glycogen synthase kinase-3 - modified cell differentiation through GFAP, BDNF, beta3 tubulin, cluster determinant 133, 44, GDNF - induced cell death through Bax, ROS, glutathione, Bcl-2 - inhibited tumor invasion through TIMP1, MMP-2, interleukin-6, NF-κB - inhibited tumor angiogenesis through VEGF - immunological damage through TGF-β1
antidementia drugs	memantine	- dementia	- breast, colon, lung, prostate, skin cancer, glioma, leukemia, neuroblastoma	- NMDA antagonism-related modification of signaling pathways, such as ERK1/2 - inhibition of tumor growth and metastases through FGFR1-4, HSPGs, GLG1, ESL-1
	galantamine		- cervical adenocarcinoma	- inhibition of tumor growth through positive allosteric modulatory effect on α7-subtype of nicotinic acetylcholine receptors

Psychiatric drugs may exert anticancer effects through many mechanisms (Table 1, Table 2 and Table 3; Figure 1). Antidepressants have been shown to exert anticancer effects through various pathways, including mitochondrial, reactive oxygen species (ROS), Ca^2+^-dependent, cell apoptosis, and signaling pathways (e.g., PI3K/AKT/mTOR). Additionally, these drugs exhibit antiproliferative effects involving cell cycle regulators, lysosomal enzymes, or growth factors (e.g., matrix metallopeptidase 9 and vascular endothelial growth factor), which are known to participate in tumor growth and metastasis (details in Table 1) [24]. Many of these mechanisms are identical to those of typical anticancer drugs [33]. Antidepressants can also inhibit tumor development by altering the immune response or the tumor cell microenvironment. Moreover, multidrug resistance in cancer cells can also be circumvented by antidepressant therapy. An example would be fluoxetine, which has been shown to enhance the antitumor effects of doxorubicin, paclitaxel, and vinblastine by inhibiting the multidrug resistance pump, ultimately increasing intracellular drug concentration in vitro [24]. Therefore, by adding antidepressants to conventional anticancer drugs, we may potentially reduce the serious side effects of those treatments [24]. The antitumor effects have been demonstrated for selective serotonin reuptake inhibitors (SSRIs, such as fluoxetine, paroxetine, sertraline, and citalopram), tricyclic antidepressants (TCAs, such as amitriptyline and imipramine), serotonin–norepinephrine reuptake inhibitors (SNRIs, such as duloxetine and St. John’s Wort), tetracyclic antidepressants such as mirtazapine, and monoamine oxidase inhibitors (MAOIs), such phenelzine [24]. The anticancer potential of antidepressants appears to be extensive, as fluoxetine alone has been shown to possess potential anticancer properties against non-small cell lung cancer, colon cancer, lymphoma, gastric adenocarcinoma, ovarian carcinoma, glioma/neuroblastoma, Burkitt lymphoma, and hepatocellular carcinoma [24]. Generally, among antidepressants, fluoxetine, amitriptyline, imipramine, mirtazapine, and St John’s Wort have the most comprehensive anticancer literature [20]. In addition to their anticancer mechanisms, antidepressants primarily improve mood and alleviate somatic symptoms such as pain, reduce anxiety and nervousness, and improve sleep, which allows for better recovery of the body fighting cancer [24]. Vortioxetine seems to be an especially interesting antidepressant drug with anticancer properties, particularly regarding its antiglioblastoma effects [23]. Its synergistic effect with current standard-of-care chemotherapies has been confirmed in vivo involving the Ca^2+^-driven AP-1/BTG-pathway and increased survival. As an antidepressant, it works via blockade of serotonin reuptake and modulates various serotonin receptors such as 5HT1A and 5HT7 [23].

It has been shown that more than 50 types of cancers may be inhibited by antipsychotic drugs, with four antipsychotics (chlorpromazine, pimozide, thioridazine, and trifluoperazine) having the largest number of anticancer reports [20] (Table 2). The scale of research on the anticancer effects of antipsychotic drugs is evidenced by the fact that chlorpromazine alone has been proven to have anticancer effects on colorectal, gingival, tongue, laryngeal, liver, lung, ovarian, pancreatic, and skin cancer, as well as on Ehrlich ascites tumor, fibrosarcoma, glioma/glioblastoma, leukemia, lymphoblastoma, mastocytoma, medulloblastoma, melanoma, and sarcoma [20]. New studies suggest that the atypical antipsychotic brexpiprazole also shows interesting anticancer potential, especially in glioblastoma [23]. Although antipsychotics at non-toxic doses have very minimal anticancer effects alone, their combination with antiproliferative medications demonstrates an enhanced antiproliferative effect compared with using each agent individually [20]. There are numerous mechanisms through which antipsychotics inhibit cancer, such as blockade/inhibition of dopamine D2 receptor, which have cytotoxic effects through activation of the integrated stress response, and through other signaling pathways (e.g., PI3K/AKT/mTOR/NF-κB, ERK, Wnt/ß-catenin, and bcl2), metabolic pathways (e.g., intracellular cholesterol transport, and glycolysis), and various proteins (e.g., transcription factors). Furthermore, they may induce autophagy and Ca^2+^-dependent signaling cascades, along with other mechanisms such as gene CD133 and SOX2 suppression, and p53 protein, among others (details in Table 2) [20,27].

In addition to antidepressants and antipsychotics, anticancer potential has been identified in mood stabilizers such as lithium and valproates [30]. Lithium studies have found effects on induced apoptosis, autophagy, inhibition of tumor proliferation, invasion, metastasis, and cell cycle arrest, as well as enhanced antitumor properties of anticancer drugs [29,33] (Table 3). Valproates can inhibit histone deacetylases (HDACs), the expression of pyruvate kinase M2 isoform (PKM2), which leads to reduced cell proliferation and colony formation, affects microRNA expression, and sensitizes tumors to chemotherapy (Table 3) [29,34]. Among antidementia drugs, the antitumor effect was found for memantine, which can regulate tumor growth, invasion, and metastasis in various cancer types through multiple mechanisms dependent on NDMA inhibition (Table 3) [35]. Although some antidementia medications from the class of acetylcholinesterase inhibitors (rivastigmine and galantamine) have been associated with an increased risk of lung cancer [36], the cytotoxic effects of galantamine against human cervical adenocarcinoma have also been observed [37]. It has also been found that cancer survivors have an 8% to 14% lower risk of dementia, while those with dementia have a 25% lower cancer risk [36]. Additionally, anticancer drugs, such as tamoxifen and chemotherapeutics, have been shown to reduce the risk of Alzheimer’s disease [38].

The anticancer effects of psychotropic drugs have been primarily explored through both in vitro and in vivo studies in animals, as well as in vitro studies on human cancer cell lines, along with a few clinical trials in humans [20,24]. In vitro studies often provide initial insights into how these drugs may affect cancer cells directly, allowing researchers to observe cellular responses, mechanisms of action, and potential therapeutic targets. In vivo studies, on the other hand, involve testing these drugs in animal models or clinical trials to evaluate their efficacy, safety, and overall impact on cancer progression in a more complex biological system. Both types of studies are crucial for understanding the potential anticancer properties of psychotropic drugs and contribute to the development of new therapeutic strategies [20,24].

The anticancer effects of psychotropic drugs can vary significantly depending on the specific drug, dose, the type of cancer being treated, and the individual patient’s response. Some studies suggest that lower doses may be effective in specific contexts because of the ability of these drugs to modulate biological pathways involved in cancer progression, while in other cases, higher doses (e.g., of risperidone) may be necessary to achieve the desired therapeutic effect [20,24,32]. However, the optimal dosing strategy is still a subject of ongoing research and can be influenced by factors such as drug metabolism, potential side effects, and interactions with other medications. Overall, the appropriate dosage for any therapeutic application should be determined by healthcare professionals based on clinical evidence, patient characteristics, and ongoing monitoring of treatment effects.

## 4. Repurposing

Drug repurposing involves using existing drugs for new therapeutic purposes, which offers a cost-effective and time-efficient strategy in medicine [18,26]. Psychiatric drugs are particularly attractive candidates in oncology because of their well-characterized pharmacokinetics and safety profiles. The overwhelming majority of research conducted to date on the anticancer properties and mechanisms of psychiatric medications is based on animal studies and human in vitro cancer cell lines (see reviews [18,20,24]). For example, an in vivo study of vortioxetine in mice showed reduced brain tumor size when measured by magnetic resonance imaging [23]. Only a few studies have examined the course of cancer in individuals taking psychiatric medications, such as quetiapine or lithium (see review [18,34]). While preclinical data are compelling [23], translating these findings into clinical practice requires rigorous trials. Several human clinical anticancer trials have been conducted so far using antipsychotic drugs such as pimozide, thioridazine, and chloropromazine [18]. These studies showed that pimozide in metastatic melanoma (phase II) achieved a partial 17% response to treatment, thioridazine in acute myeloid leukemia (phase I) reduced blast levels by 55%, while the addition of chlorpromazine to temozolomide in patients suffering from glioblastoma with unmethylated O6-methylguanine methyltransferase gene promoter (phase II) led to longer progression-free survival time [18,39]. Of antidepressive drugs, tranylcypromine (in leukemia, glioblastoma, and head and neck squamous cell carcinoma), phenelzine (in prostate cancer), but not desipramine, demonstrated anticancer therapeutic effects in clinical trials (phase I/II) [31]. Randomized phase II studies have also confirmed the antineoplastic effect in hematological/solid cancer patients and improved median overall survival of valproates when concomitantly administered with radio/chemotherapy. Partial responses have been observed in lithium studies in glioblastoma, leukemia, and thyroid cancer [34]. Of antidementia drugs, two clinical trials of memantine for glioblastoma have been registered, along with one for colon cancer [35].

## 5. Limitations

The main limitation of this study is that the results are based primarily on animal studies and in vitro studies on cancer cell lines. As described above, there are only a few studies on the anticancer effects of psychiatric medications based on human clinical trials. The formal approval process for psychotropic drugs requires safety testing for carcinogenicity in animals. In addition to their anticancer effects, psychiatric drugs have also been associated with carcinogenicity in preclinical animal studies [28]. A systematic review of databases for psychotropic drugs showed that new generation antipsychotics (90% of 10 drugs) and anticonvulsants (85% of 7 drugs) had the most evidence of carcinogenicity among the psychotropic drugs evaluated [28]. This was followed by antidepressants (63% of 11) and benzodiazepines/sedative–hypnotics (70% of 10), and finally stimulants (25% of 4 drugs). They concluded that overall, 71% of all drugs tested (30 of 42) showed evidence of carcinogenicity in 43% (38 of 88) of the detailed experimental studies in animals, but these animal-based results were not sufficient to draw definitive conclusions in humans [28]. Such discrepancies in research regarding the anticarcinogenic and carcinogenic effects of psychiatric drugs on cancer arise from several reasons [28,40]. Carcinogenicity observed in animal studies may not translate directly to humans, as biological responses may differ between species. Patients with severe mental illness often have other risk factors for cancer, such as smoking, obesity, and other comorbidities, making it difficult to isolate specific drug effects. The same drugs that demonstrate some potential for harm in one context may have therapeutic anticancer properties in another. Furthermore, even if some antipsychotics have shown carcinogenic properties among rodents, their antiproliferative properties may be useful in multidrug augmentation strategies in various cancer treatments. Additionally, part of the carcinogenic effect may result from indirect effects on metabolism and inflammation (e.g., olanzapine and liver cancer), hormonal balance, and gender (e.g., neuroleptics and pituitary/breast cancer) [28,40]. Some studies available in the literature also describe a lack of inhibitory effects of psychiatric medications, such as chlorpromazine, clozapine, flupentixol, fluphenazine, pipamperone, remixipride, risperidone, sulpiride, and trifluoperazine, on cancer (see review [20]). This effect may be partially explained by too low doses of the medications used, as in the cases of haloperidol, iloperidone, quetiapine, and thioridazine [20].

## 6. Future Directions

A significant challenge in future research on the use of psychiatric medications in cancer patients is to clarify the impact of treating psychiatric disorders on neoplastic processes. It is crucial to clarify the potential carcinogenic effects of psychiatric medications, which have been observed in preclinical animal studies. If these studies prove to be truly harmless to humans, clinical trials of psychiatric medications in cancer should determine which psychiatric medications are most effective in specific cancers. Subsequently, these studies should determine optimal dosing regimens for psychiatric and anticancer medications that balance anticancer efficacy with psychiatric effects. Understanding the interactions of psychiatric medications with standard chemotherapeutic agents and identifying biomarkers to predict which patients may benefit from such therapies appear to be key steps in this future research. The potential impact of psychiatric medications on other oncological procedures, such as radiotherapy or surgery, also may not be overlooked.

## 7. Discussion

Over the past two decades, a growing body of experimental evidence has pointed to the promising role of psychiatric drugs in cancer treatment [18,19,20,21,22,23,24,25,26]. The anticancer potential of psychiatric drugs represents an exciting interdisciplinary frontier in psychiatry and oncology. As research progresses, these medications may become valuable adjuncts in cancer therapy, offering new hope for patients with difficult-to-treat malignancies. A special place seems to belong to antidepressant vortioxetine in glioblastoma treatment, because of its significant ex vivo efficacy observed in 75% of patients [23]. In general, psychiatric medications seem to play a triple role in the treatment of cancer patients, treating psychiatric conditions associated with cancer, such as mood disorders, anxiety, psychosis, and delirium, but also directly inhibiting tumor growth, metastasis, and increasing tumor sensitivity to chemotherapeutic agents/radiotherapy [7,20,24]. Psychiatric medications have a long history of clinical use and a tolerable safety profile [26]. However, the mechanisms underlying anticancer effects are not fully understood, and more clinical work is necessary to draw definitive and real conclusions. Despite the growing body of preclinical evidence, few early phase I/II clinical trials on the anticancer efficacy of psychiatric drugs have been conducted to date [18,19,31,34,39]. One potential reason for this is that these drugs, especially antipsychotics, have significant side effects such as extrapyramidal symptoms, sedation, and anticholinergic effects, which may not be optimal for cancer patients with increased morbidity [7,20]. Therefore, more preclinical and clinical studies with antidepressants, first- and second-generation antipsychotics, mood stabilizers, and antidementia drugs are needed to clarify the precise role of these medications as repositioning agents in cancer treatment in order for safe conclusions to be reached. Further research and clinical trials are necessary to fully understand the carcinogenic and anticancer effects of psychiatric medications, their dose-dependent effects, and mechanisms of action. Although the results of preclinical studies are important, the overall clinical picture in patients with mental disorders, particularly with respect to cancer risk, is complex and requires further investigation.

## 8. Conclusions

While psychiatric medications play a significant role in the treatment of mental disorders in cancer patients, they can also be crucial in the treatment of oncology patients because of their ability to inhibit tumor growth and metastasis. They can be particularly useful between chemotherapy and radiotherapy sessions, inhibiting the proliferation and metastasis of cancer cells. However, the decision to use psychiatric medications should include a thorough risk–benefit assessment for each patient.

## Abbreviations

The following abbreviations are used in this manuscript:

AMPK	AMP-activated protein kinase
BAX	Apoptosis regulator protein
Bcl2	B-cell lymphoma 2 protein
BBB	Blood–brain barrier
BDNF	Brain-derived neurotrophic factor
BPNT1	Bisphosphate 3-primenucleotidase
Ca^2+^	Calcium ion
CBT	Cognitive–behavioral therapy
CD133	antigen (prominin-1) encoded by the PROM1 gene
CREB	Camp-Response-Element-Binding Protein
c-Myc	A family of regulator genes
CYP2D6	Cytochrome P450 2D6
Eag1	Ether-à-go-go1 K^+^ channel
ERK	Extracellular signal-regulated kinases
ESL-1	Cysteine-rich FGF receptor and E-selectin-ligand 1
FGFR	Fibroblast growth factor receptor
GDNF	Glial cell line-derived neurotrophic factor
GFAP	Glial fibrillary acidic protein
GLG1	Golgi glycoprotein 1
GSK-3	Glycogen synthase kinase-3
HDAC	Histone deacetylase
HSPGs	Heparan sulfate proteoglycans
IFN	Interferon
IL	Interleukin
IMCP	Individual meaning-centered psychotherapy
JNK/c-Jun	c-Jun N-terminal kinases
K-Ras	Kirsten rat sarcoma virus protein
KLF5	Krüppel-like factor 5
MBCT	Mindfulness-Based Cognitive Therapy
MDM2	Mouse Double Minute 2 protein
MMP-9	Matrix metallopeptidase 9
mTOR	The mammalian target of rapamycin
MYCN	N-myc proto-oncogene protein
NF-κB	Nuclear factor kappa-light-chain-enhancer of activated B cells
NMDA	N-methyl-D-aspartate
NRF2	Nuclear factor erythroid 2-related factor 2
NUPR1	Nuclear Protein 1-transcriptional regulator
p53	Tumor protein p53
PAK 4	Serine/threonine-protein kinase 4
PI3K/AKT	Phosphoinositide 3-kinase signaling pathway
PKM2	Pyruvate kinase M2 isoform
REST	Repressor element 1 silencing transcription factor
ROS	Reactive oxygen species
SIRT1	Silent mating type information regulation homolog for Sirtuin
SNAI1	Zinc finger protein
SMI	Severe mental illness
SOX2	Single-exon nuclear transcription factor 2
STAT3	Signal transducer and activator of transcription 3
TCTP	Translationally controlled tumor protein
TGF	Transforming growth factor
TIMP1	Tissue Inhibitor of Metalloproteinases 1
TORC1	Transducer of regulated CREB activity 1
VEGF	Vascular endothelial growth factor
Wnt	Wingless-related integration site
